# Clinical evaluation of laparoscopic surgery combined with triptorelin acetate in patients with endometriosis and infertility

**DOI:** 10.12669/pjms.345.15574

**Published:** 2018

**Authors:** Huiling Xue, Meiyun Liu, Wanjiao Hao, Ye Li

**Affiliations:** 1 Huiling Xue, Department of Reproductive Medicine, Affiliated Hospital of Hebei University Hebei Sheng, China; 2 Meiyun Liu, Department of Reproductive Medicine, Affiliated Hospital of Hebei University Hebei Sheng, China; 3 Wanjiao Hao, Department of Reproductive Medicine, Affiliated Hospital of Hebei University Hebei Sheng, China; 4 Ye Li, Department of Reproductive Medicine, Affiliated Hospital of Hebei University Hebei Sheng, China

**Keywords:** Endometriosis, Infertility, Laparoscopic surgery, Pregnancy rate, Pregnancy outcome, Clinical effect, Adverse reactions

## Abstract

**Objective:**

To investigate the effects of laparoscopic surgery combined with triptorelin acetate, gestrinone and mifepristone on pregnancy rate, pregnancy outcome, long-term recurrence and adverse reactions in patients with endometriosis (EMT) complicated with infertility.

**Methods:**

In this study, 150 patients with EMT and infertility were selected and randomly divided into triptorelin group (group A), gestrinone group (group B) and mifepristone group (group C), with 50 people in each group. Treatment was with gonadotropin-releasing hormone (GnRH-a) after laparoscopic surgery.

**Results:**

The success rate of pregnancy in group A was higher than that in group B and C. The incidence of adverse pregnancy outcome was lower than that in group B and C. Kupperman score and subjective symptom score were lower than those in group B and C before treatment. Sex hormone level and CA125 level were significantly better. In the B and C groups and before treatment; the long-term recurrence rate was significantly lower than the B and C groups; the incidence of adverse reactions was similar.

**Conclusion:**

Laparoscopic surgery combined with GnRH-a can improve the success and good rate of pregnancy in EMT patients, reduce clinical symptoms, avoid long-term recurrence, and increase the risk of adverse reactions. Triptorelin acetate is superior to gestrinone and mifepristone.

## INTRODUCTION

Endometriosis (EMT) is a common benign disease of childbearing age. The incidence is increasing year by year. The clinical manifestations are pain, infertility and other symptoms.^1^ Patients with infertility often bring great pressure to themselves and their families. Therefore, improving the cure rate of EMT combined with infertility patients is the goal pursued by the majority of reproductive doctors. The specific pathogenesis of infertility caused by EMT has not been fully elucidated. Domestic and international studies have concluded that infertility caused by EMT is related to pelvic anatomical factors, immune factors, pelvic microenvironment and ovarian function.^2^

Some studies have also suggested that macrophage proliferation, activation and phagocytosis in pelvic fluid of EMT patients, combined with the release of a large number of inflammatory factors, growth factors and angiogenic factors, further affect sperm transport and sperm-egg binding.^3^ Various studies shows that endometrial antibodies in EMT patients increase, leading to abnormal endometrial metabolism, menstrual cycle disorders, and thus infertility.^4^ A large number of studies have shown that simply expecting treatment or drug treatment cannot increase the pregnancy success rate of patients with EMT and infertility, and the adverse effects of using the drug itself alone will affect the patient’s fertility.^5^,^6^

In the 2014 European Society for Human Reproduction and Embryology (ESHRE) Guidelines:^7^ Treatment of Endometriosis, for patients with EMT with infertility, laparoscopic adhesions should be performed (focal cauterization or Resection), and on the basis of surgical treatment to give medication, but cannot simply give expectation treatment or medication to improve pregnancy success rate and reduce recurrence. It is generally believed that the treatment principle of EMT patients is to reduce the lesions, reduce pain, promote fertility, prevent and reduce recurrence.^8^ With the continuous development of minimally invasive techniques, laparoscopic techniques have made great progress in the treatment of EMT, and laparoscopic surgery combined with drug therapy has been the gold standard for EMT.^9^

At present, the combination of laparoscopic drugs is gonadotropin-releasing hormone (GnRH-a), gestrinone, mifepristone, etc.^10^ Studies have shown that laparoscopic adjuvant medication can effectively avoid long-term recurrence of EMT patients, improve the chance of pregnancy,^11^ but currently there is no conclusion on the choice of drug types. The effect of postoperative combination of the above three drugs on pregnancy success rate and pregnancy outcome in patients with EMT combined with infertility has rarely been reported at home and abroad.

In this study, 150 patients with EMT and infertility were treated with laparoscopic surgery. The patients were treated with triptorelin acetate, gestrinone tablets and mifepristone tablets. The drug treatment and pregnancy rate were analyzed. The relationship between pregnancy outcomes; Kupperman score, subjective symptom score, sex hormone level, CA125 level, long-term recurrence rate and adverse reactions before and after treatment with three drugs.

## METHODS

One hundred fifty cases of EMT combined with infertility patients were admitted to the Affiliated Hospital of Hebei University from January 2013 to January 2016. These enrolled patients aged 21 to 42 years, (27.51 ± 5.12) on average. The course of disease was 2 to 10 years, (4.81 ± 2.92) on average. The duration of infertility was one to six years, with an average of (3.15 ± 1.23). The menarche age was 12 to 15 years, and the average age was (13.82 ± 1.15) years.

### Inclusion criteria

1. Complies with the diagnostic criteria for the diagnosis and treatment of endometriosis.^12^
2. Diagnosed by laparoscopy and pathological biopsy
3. Infertility >1 year with normal sexual life
4. Family members sign informed consent.

### Exclusion criteria

1. Infertility caused by immune factors or other factors of the body
2. Genital malformations, combined malignant ovarian tumors, pelvic inflammatory masses, and adenomyosis
3. Infertility caused by semen abnormalities
4. Taboo for the application of research drugs
5. A history of pelvic surgery
6. Hemorrhagic disease
7. Important organ dysfunction
8. Pregnant lactating women
9. Patients who do not agree to participate in the study
10. Incomplete clinical data.

The randomized and single-blinded method was used to divide the patients into the Triptorelin group (Group-A), the gestrinone group (Group-B), and the mifepristone group (Group-C) (n=50). There was no statistical difference in age, the course of disease, staging by the Retrospective American Fertility Association (r-AFS) and disease classification (Table-I).

**Table-I:** Baseline clinical data (x ± s) (n).

Group	Case No. (n)	Average age (Year)	Average disease course (Year)	Infertility course (Year)	Average menarche age (Year)	Pathological type (Ovary/peritoneum / deep infiltration)	r-AFS stage (I/II/III/IV)
A	50	26.71±4.82	4.39±2.45	3.09±1.45	13.82±1.15	16/25/9	15/20/8/7
B	50	27.51±5.12	4.81±2.92	3.55±1.23	13.55±1.23	15/25/10	16/21/8/5
C	50	26.12±4.90	4.26±2.89	4.02±1.29	12.02±1.29	14/27/9	16/20/8/6

### Surgical methods

Under general anesthesia with tracheal intubation, the pneumoperitoneum was established, the pressure was 11~13mmHg, the patient was placed in the lithotomy position, and the Trocar was placed in a four-hole method. The pelvic cavity was fully examined, and the adhesion lysis was performed. The normal pelvic anatomy was restored and the placement was transparent. Sodium carbonate prevents adhesion, suture incision, pathological tissue inspection.

### Drug therapy

**Group-A:** Triptorelin acetate (Commodity name: Diphereline, IPSENPHARMA, 3.75 mg/dose, National Medicine Permit No. H20140298). Intramuscular injection started on the 5th day of the first menstrual onset after surgery, 3.75 mg, once a month, for three consecutive months.

**Group-B:** Gestrinone capsule (Huarun Zizhu Pharmaceutical Co., Ltd., National Medicine Permit No. H19980020, 2.5mg/ capsule). Oral administration started on the 5th day of the first menstrual onset after surgery, 2.5 mg, twice a week for six consecutive months.

**Group-C:** Mifepristone tablets (Beijing Keyi Biotechnology Development Co., Ltd., National Medicine Permit No. H20084624, 25 mg/tablet). Oral administration started on the 5th day of the first menstrual onset after surgery, 25 mg, twice a day for three consecutive months.

### Observation indices

The success rate of pregnancy within two years after surgery was followed up; 2) the pregnancy outcomes of the successfully pregnant patients were recorded; 3) Kupperman score and subjective symptom score were used to assess the severity of disease. Kupperman scores mainly include hot flashes, sweat, perceptual abnormalities, insomnia, impatience, depression, dizziness, tiredness, fatigue, muscle pain, joint pain, headache, heart palpitation and skin formication, with a total score of 63 points.^13^ Subjective symptom scores mainly include hot flashes and night sweat (4 points), vulva (2 points), vaginal discomfort (2 points), insomnia (2 points), and other items (1 point). The score for each item was 0 to three points. The total score = score of classification × degree score; the higher the score, the heavier the condition. The detection of sex hormone indicators such as follicle stimulating hormone (FSH), estradiol (E2), luteinizing hormone (LH) and CA125 were tested using Roche COBAS INTEGRA 800 automatic biochemical analyzer. The patients were followed up postoperatively for two years to record the number of recurrence and percentage. The number of cases and percentage of adverse reactions during treatment were observed, including low sexual desire, abnormal bone density, abnormal vaginal bleeding, and liver function damage.

### Statistical analysis

All data were analyzed by SPSS 22.0. The categorical data were expressed as x ± s. Intra-group comparisons were performed by repeated measurement analysis of variance, and inter-group comparisons were conducted by the paired t-test. The numerical data were expressed as percentage (%), and inter-group comparisons were carried out by the χ^2^ test. P<0.05 was considered statistically significant.

## RESULTS

### Pregnancy success rates

The pregnancy success rate of 150 patients was 53.33% (80/150). The result of Group-A was significantly higher than those of Group-B and Group-C (P<0.05) (Table-II).

**Table-II:** Pregnancy success rates and outcomes [case (%)].

Group	Case No. (n)	Pregnancy success rate	Good outcome			Poor outcome		

		n (%)	Natural childbirth n (%)	Caesarean section n (%)	Total incidence (%)	Ectopic pregnancy n (%)	Spontaneous abortion n (%)	Total incidence (%)
A	50	40 (80)	26 (52)	12 (24)	76	0	2 (4)	4
B	50	24 (48)*	12 (24)	6 (12)	36*	1 (2)	5 (10)	12*
C	50	16 (32)*	9 (18)	1 (2)	20*	1 (2)	5 (10)	12*

* Compared with Group-A, P<0.05.

### Pregnancy outcomes

The incidence of adverse pregnancy outcomes of Group-A (4.00%) was significantly lower than those of Group-B (12.00%) and Group-C (12.00%, P<0.05) (Table-II).

### Kupperman scores and subjective symptom scores

The Kupperman score and subjective symptom scores after treatment of Group-A were lower than those of Group-B and Group-C, and before treatment, among which the differences were statistically significant (P<0.05) (Table-III).

**Table-III:** Kupperman scores and subjective symptom scores (x ± s).

Group	Case No.	Kupperman score		Subjective symptom score	

		Before treatment	After treatment	Before treatment	After treatment
A	50	14.87±2.65	8.43±1.45#	9.71±1.54	2.58±0.57#
B	50	14.94±2.68	11.43±1.90*#	9.77±1.58	5.02±0.99*#
C	50	14.88±2.61	11.41±1.85*#	9.73±1.49	5.99±0.82*#

Compared with Group-A,

* P<0.05; compared with scores before treatment,

# P<0.05.

### Sexual hormone and CA125 levels

The levels of sex hormones and CA125 in Group-A were significantly better than those of Group-B and Group-C, and before treatment (P<0.05) (Table-IV).

**Table-IV:** Sexual hormone and CA125 levels (x ± s) (n=50).

Group	FSH (U/L)		E2 (ng/L)		LH (U/L)		CA125 (U/ml)	

	Before treatment	After treatment	Before treatment	After treatment	Before treatment	After treatment	Before treatment	After treatment
A	5.66±1.29	2.40±0.67#	180.96±24.78	96.81±9.75 #	6.82±1.53	2.52±0.61 #	54.71±8.44	24.18±4.30 #
B	5.52±1.25	3.94±0.95*#	182.77±25.43	154.42±18.03*#	6.71±1.49	3.94±0.95*#	53.53±8.29	36.86±6.38*#
C	5.81±1.20	3.89±0.71*#	180.23±24.93	157.52±17.89*#	6.81±1.51	3.89±0.71*#	55.21±8.32	35.84±6.91*#

Compared with Group-A,

* P<0.05; compared with scores before treatment,

# P<0.05.

### Long-term recurrence rates

The long-term recurrence rate of Group-A was significantly lower than those of Group B and Group-C (P<0.05) (Table-V).

**Table-V:** Long-term recurrence rates [case (%)].

Group	Case No.	1 year after surgery	2 years after surgery	Total recurrence rate
A	50	1 (2)	0	1 (2)
B	50	4 (8)*	2 (4)*	6 (12)*
C	50	4 (8)*	2 (4)*	6 (12)*

* Compared with Group-A, P<0.05.

### Incidence of adverse reactions

The three groups had similar incidence rates of adverse reactions (P>0.05) (Table-VI).

**Table-VI:** Incidence rates of adverse reactions.

Group	Case No.	Hyposexuality	Bone mineral density abnormality	Abnormal vaginal bleeding	Liver dysfunction	Total adverse reactions
A	50	1 (2)	0	1 (2)	2 (4)	4 (8)
B	50	0	1 (2)	1 (2)	1 (2)	3 (6)
C	50	1 (2)	0	2 (4)	1 (2)	4 (8)

Compared with Group-A, *P<0.05.

## DISCUSSION

Because laparoscopic minimally invasive surgery cannot completely remove small, deep and atypical lesions in EMT patients, residual lesions can recur in a short period of time under hormone stimulation, seriously affecting clinical efficacy and treatment compliance.^14^ In recent years, the importance of drug-assisted treatment after laparoscopic surgery in EMT patients has been widely confirmed. Among them, GnRH-a and oral contraceptives are the most commonly used, but the comparative study on the efficacy and safety of different drugs is still lacking. There is some controversy.^15^ Laparoscopic combined drug application mainly through the regulation of postoperative ovarian and pituitary function, induced endometrial atrophy and amenorrhea, promote the absorption of residual foci and play a therapeutic role. Triptorelin acetate is a natural GnRH isoform heterogeneous body. It is a kind of synthetic decapeptide compound, which can promote pituitary inhibition of follicle estrogen FSH, luteinizing hormone LH release, inhibit pituitary secretion of gonadotropin, leading to ovarian hormone. The level drops and temporary amenorrhea occurs.^16^ It is used for the pharmacological effects of laparoscopic surgery in patients with EMT combined with infertility, mainly relying on its two-way regulation of pituitary, which significantly reduces the secretion of ovarian-related hormones on the one hand, stimulates the apoptosis of endometrial cells and disappears of residual lesions; On the other hand, it has an up-regulation effect on the growth of helper T lymphocytes, further enhancing the immune system function and improving the body’s own killing effect on residual lesions.^17^,^18^ Studies have confirmed that EMT patients treated with GnRH-a reverse addition therapy can improve the clinical symptoms, balance of sex hormones, and the exact effect.^19^,^20^ Studies have also shown that the combination of triptorelin and related receptors is 120 to 180 times that of native GnRH, and its activity is 30 to 50 times.^21^

Gestrinone is a 19-nortestosterone steroidal drug with antiprogestin, moderate antiestrogens and resistant gland effects, which increases free testosterone levels, reduces sex hormone binding protein levels, inhibits FSH, LH peaks and reduces LH The mean value causes the estrogen level in the body to decrease, and the ectopic endometrium shrinks and absorbs. At the same time, gestrinone also has effects on the hypothalamic-pituitary neuroendocrine axis, down-regulation of gonadotropin release and estrogen and progesterone hormone secretion levels, which is of great significance for stimulating ectopic endometrial cell apoptosis.^22^ The above two effects can play a synergistic effect in accelerating the disappearance of residual endometriotic lesions and avoiding long-term recurrence. Mifepristone is an antiprogestin drug with a strong antiprogestin effect, which can cause amenorrhea in the body and atrophy of ectopic lesions. Its mechanism of action is similar to gestrinone.

The results of this study, the success rate of laparoscopic combined with hormone therapy in 150 patients was 53.33% (80/150). The proportion of adverse outcomes in 80 patients with successful pregnancy was 30% (24/80), which was significantly higher than that in normal women of childbearing age. The results were consistent with the results of the domestic survey.^23^ In this study, the success rate of pregnancy in Group-A was significantly higher than that in Group B and C, and the incidence of adverse pregnancy outcomes in Group-A was significantly lower than that in the other two groups. The reason may be that triptorelin can inhibit endogenous LH secretion, effectively prevent follicle hyperluteinization, promote follicle developmental synchronization, and improve the quality of follicles.^24^ Similar to the results of this study. The other two groups of drugs lack such efficacy. The Kupperman score and subjective symptom scores after treatment in Group-A were significantly lower than those in Groups B and C and before treatment, confirming that the use of GnRH-a after laparoscopic surgery may help in improving the related symptoms of EMT patients. The level of CA125 is a sensitive indicator for the severity of EMT, with a positive correlation.^25^

In this study, the level of sex hormones in group A was significantly better than that in group B. In group C, the levels of sex hormones in group three were better than those before treatment. The level of CA125 was significantly decreased. It suggests that triptorelin has obvious advantages in regulating sex hormones and CA125 levels for EMT patients with infertility. The long-term recurrence rate of the patients in Group-A was significantly reduced, indicating that GnRH can not only regulate the level of sex hormones, but also is one of the key mechanisms to optimize short- and long-term clinical curative effects. There was no significant difference in the incidence of adverse reactions in all patients, suggesting that the adjuvant therapy with triptorelin did not induce an increase in the incidence of adverse reactions in EMT patients and aggravation, which conforms the safety needs of clinical treatment.

## CONCLUSION

In conclusion, combined GnRH-a after laparoscopic surgery can improve the success rate and good efficacy rate of pregnancy, effectively alleviate clinical symptoms, improve the level of sex hormones and CA125, avoid long-term recurrence, without increasing the risk of adverse reactions. Triptorelin acetate has better effects than gestrinone and mifepristone.

### Authors’ Contributions

**HX & ML** designed this study and prepared this manuscript.

**HX, WH & YL** performed this study.

**WH & YL** collected and analyzed clinical data.
